# Early embryonic exposure of freshwater gastropods to pharmaceutical 5-alpha-reductase inhibitors results in a surprising open-coiled “banana-shaped” shell

**DOI:** 10.1038/s41598-019-52850-x

**Published:** 2019-11-11

**Authors:** Alice Baynes, Gemma Montagut Pino, Giang Huong Duong, Anne E. Lockyer, Carmel McDougall, Susan Jobling, Edwin J. Routledge

**Affiliations:** 1 Institute of Environment, Health and Societies, Brunel University London, Uxbridge, UB8 3PH United Kingdom; 2 Centre for Obesity Research, Division of Medicine, University College London (UCL), 5 University Street, London, WC1E 6JF United Kingdom; 3 Australian Rivers Institute, Griffith University, 170 Kessels Road, Nathan, QLD 4111 Australia

**Keywords:** Endocrinology, Body patterning, Embryogenesis

## Abstract

In vertebrates, the steroidogenesis enzyme 5α-reductase converts testosterone to the more potent androgen 5α-dihydrotestosterone. Homologues of 5α-reductase genes have been identified in molluscs. However, recent findings suggest that vertebrate-type steroid androgens are not utilised in molluscan reproductive development. Genomic searches have revealed that molluscs do not possess many of the steroidogenic enzymes required to make testosterone, nor a nuclear androgen receptor. Consequently, the role of 5α-reductase in molluscs presents a mystery. Here, developmental exposures of *Biomphalaria glabrata* to selective pharmaceutical 5α-reductase inhibitors elicited a strong, highly reproducible phenotypic response characterised by the development of elongated “banana-shaped” shell morphology. In comparison to untreated snails, the shells are open-coiled and the whorls are unattached. Dutasteride (5α-reductase inhibitor) is approximately 10-times more potent at provoking the banana-shaped shell phenotype than finasteride, paralleling the pharmaceuticals’ efficacy in humans. Other enzyme inhibitors with different modes of action were tested to investigate the specificity of the phenotype. However, only the pharmaceutical 5α-reductase inhibitors provoked the response. Dutasteride elicited the same phenotype in a second gastropod, *Physella acuta*. In the absence of evidence for *de novo* androgen steroidogenesis in molluscs, these findings suggest that novel substrates for 5α-reductase exist in gastropods, lending support to the contention that molluscan endocrinology differs from the well-characterised vertebrate endocrine system.

## Introduction

It is frequently assumed that homologous genes found in different species will perform homologous functions^1^. The recognised function, or role, of a gene, is often based on its observed function in the organism from which it was first characterised; usually a model organism and often a mammal. However, when making comparisons between phyla, this widely-held view could lead to incorrect assumptions about an organism’s biology. One prime example of this is the view that molluscs use vertebrate sex steroids, such as testosterone, in their reproductive endocrinology^2–6^. Currently this is an area of controversy, with some researchers measuring vertebrate sex-steroids in molluscan tissues and equating these values to *de novo* synthesis and a physiological role^7–12^. Whereas other authors counter that although these hormones have been detected in molluscan tissues they are not endogenous and suggest they are merely absorbed from the environments they live in, and do not play a role in gametogenesis or reproduction^13–20^.

Molluscs make up a large and diverse phylum^21^, second only to Arthropoda in the number of species. They are vital components of most ecosystems and are of great economic significance across the world both in terms of their value in aquaculture (e.g. oysters); their impact as agricultural pest species (slug and snail damage) and as intermediate hosts for parasites (transmitting flukes and trematodes). Therefore, the pursuit of a comprehensive understanding of molluscan endocrinology is important.

### The case of 5α-reductase in vertebrate, molluscan and plant endocrinology

**Figure 1:**
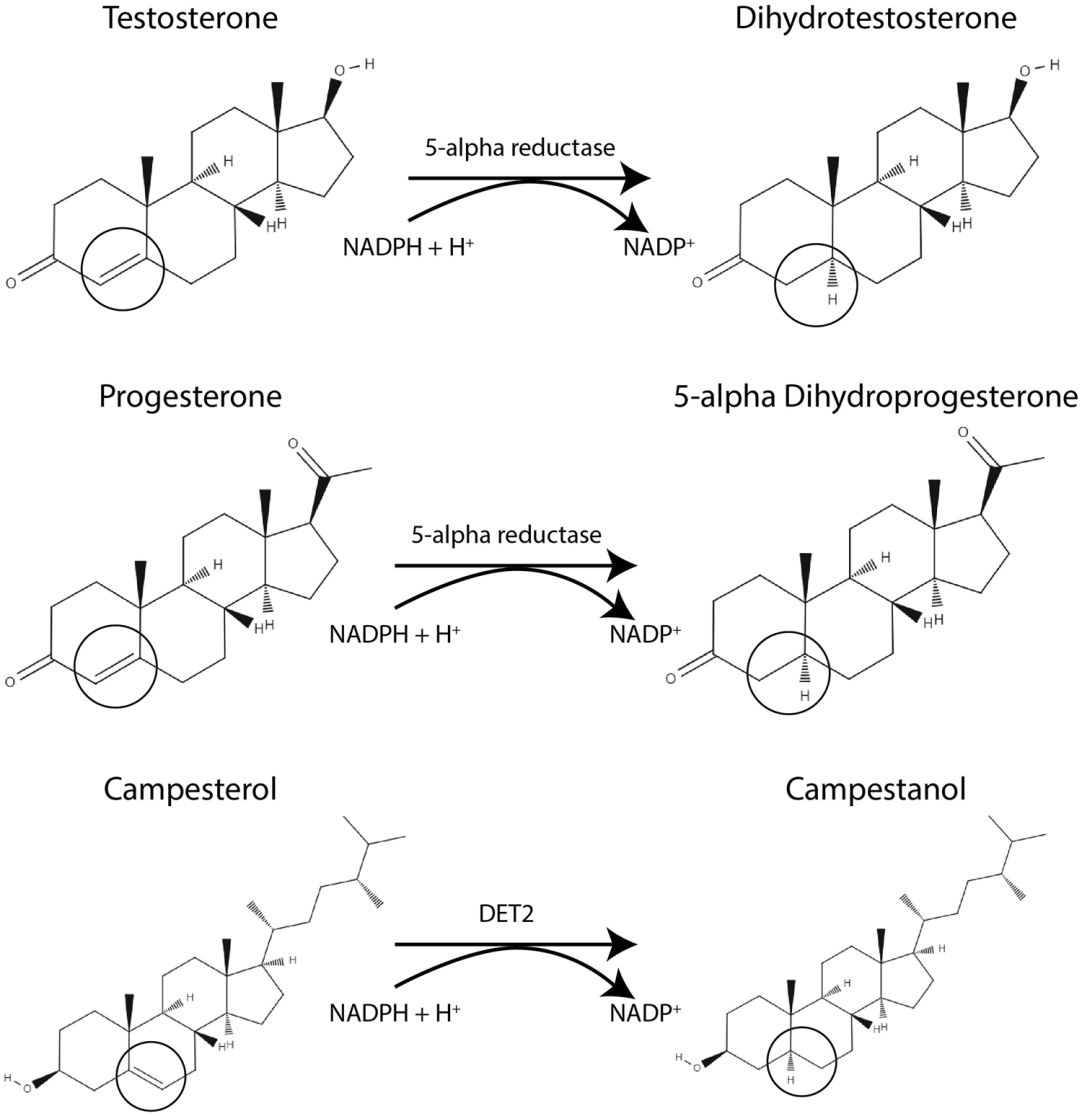
The steroidogenic enzymes, 5αR or DET2, convert a range of steroid hormones. For example, here the structures of two vertebrate steroids, testosterone and progesterone, and one plant brassinosteroid (campesterol) substrate and resulting products are shown. In each instance, the oxidoreductases enzyme (5αR or DET2) converts the Δ^4,5^ or Δ^5,6^ double-bond (circled) to a single bond (circled)^39,57,58^.

The steroid enzyme 5α-reductase (5αR) is known to be vital in male vertebrate sexual development and reproductive health^22^. In vertebrates, 5αR converts the main circulating steroid androgen, testosterone (T), to the more potent 5α-dihydrotestosterone (DHT) form (Fig. 1). It is also known to convert several other steroids including progesterone into 5α-dihydroprogesterone (Fig. 1), cortisol into 5α-corticosterone and androstenedione into 5α-androstenedione. However compared to DHT, the biological activity of other 5α reduced vertebrate steroids are less well known. Two 5αR enzymes have been studied for many years, namely 5αR1, 5αR2. These enzymes can convert T into DHT *in vitro*, although with different kinetics (reviewed in^23^). They also have different patterns of tissue expression with 5αR1 being located mainly in non-androgen target tissues (e.g. skin), whereas 5αR2 has more often been reported to be expressed in androgen-target tissues (e.g. prostate)^24^. A third 5αR (5αR3) has recently been identified^25^, and is separated from 5αR1 and 5αR2 by phylogenetic analysis^23^. 5αR3 has been reported to be almost ubiquitously expressed in all tissue, however, its exact function is less well understood (reviewed in^23^).

In humans, genetic mutations of the 5αR2 can result in deficient DHT levels during development, leading to a condition of male pseudohermaphroditism. These children are born with ambiguous genitalia and fail to develop male patterns of facial and body hair growth and secondary sex characteristics^22^. DHT, and thus 5αR, is also vital for prostate development^26^, however excessive DHT in older men is thought to be a driver of benign prostate hyperplasia and some forms of prostate cancer. This has led to the development of potent pharmaceutical 5αR inhibitors, including finasteride (FIN) and dutasteride (DUT), to reduce DHT production (e.g.^27,28^). These inhibitors also result in altered sexual development and/or reproductive development in other vertebrates such as fish, lizards and frogs, which also possess 5αR1 and 5αR2 homologues^23,29,30^, suggesting a common function in vertebrates generally.

5αR homologs have also been identified in a range of invertebrate groups including molluscs (e.g. bivalves: *Crassostrea hongkongensis*^31^ and gastropods: *Biomphalaria glabrata*^32^). Experiments exposing molluscan tissues/whole adult bivalves to exogenous radio-labelled testosterone have reported conversion into DHT^8,16^. Similar experiments using radio-labelled androstenedione in echinoderms resulted in conversion to 5α-androstanedione^33^. Moreover, exposure of sea urchin tissue (or whole adult) to radio-labelled androstenedione and a 5αR inhibitor (finasteride) together, reduced the rate of conversion^34^, suggesting that finasteride acted on the site of androstenedione conversion. This evidence supports the notion that vertebrate and invertebrate 5αRs are functional orthologues.

Many authors have also measured vertebrate hormones, such as testosterone, in tissue of molluscs sampled in the wild (reviewed in^35^). This has led to the assumption that molluscs, like vertebrates, produce testosterone and DHT *de novo*, and that these steroids are involved in gametogenesis and reproduction. However, our recent work suggests the freshwater gastropod *B. glabrata* does not use vertebrate steroid androgens in their reproductive development^20^ and this finding has been supported by number of recent investigations of molluscan genomes (*B. glabrata*, *Lottia gigantea* or *Crassostrea gigas*) where no evidence for a steroid androgen receptor (AR) homologue has been found^36,37^. Indeed the absence of the whole Group 3 C nuclear receptor family, which includes the AR, progesterone receptor (PR) or glucocorticoid receptor (GR), and the mineralocorticoid receptor (MR), suggest it is unlikely these steroids are functional in molluscs^36^. Genomic searches have also shown that molluscs do not possess the cholesterol side-cleavage enzymes (CYP11A) vital for vertebrate sex hormone steroidogenesis^32,38^.

Interestingly, 5αR homologs have also been identified in a range of other organisms, including plants^39^. In plants, the DET2 gene is involved in brassinolide steroidogenesis by catalysing the conversion of campesterol to campestanol^39^ (Fig. 1). Studies have shown that in experimental conditions DET2 can also convert testosterone to DHT^39,40^ and that human steroid 5αR expressed in det2 mutant plants can substitute for DET2 in brassinosteroid biosynthesis^39^. This evidence supports DET2 also being a functional ortholog of 5αR in plants. However, unlike in the molluscan and echinoderm models, it is not assumed that plants use vertebrate androgens. Instead, it has led to a more complete understanding of plant steroidogenesis, and the vital role of brassinosteroids (plant steroids) in plant growth, development, and reproduction^39–43^. Similarly, discovering the role of 5αR in molluscs may be the first step towards revealing molluscan endocrinology.

The aim of this study was to (i) determine if 5αR homologues are present in the early development of the gastropod mollusc *Biomphalaria glabrata*, (ii) to investigate if 5αR homologues perform a physiological role in early development of gastropod molluscs using selective pharmaceutical 5αR inhibitors (DUT, FIN) as tools, and (iii) to determine if the potencies of these inhibitors reflect those seen in vertebrates. To ascertain if any observed effects of 5αR inhibitors were specific to this group of compounds, we also tested a variety of reported steroidogenic enzyme inhibitors, a mutagen, an anti-inflammatory chemical, and compounds previously reported to affect shell coiling and chirality in molluscs.

## Results and Discussion

### 5α-reductase genes and proteins in *B. glabrata* embryos

Homologs of both 5αR1 and 5αR2 genes were previously identified in the *B. glabrata* genome^32^ and expression detected in several *B. glabrata* adult tissues, such as the mantle edge, hepatopancreas, and the kidney^32^. Here we can confirm that both 5αR1 and 5αR2 genes are also expressed in *B. glabrata* embryos. However, qPCR quantification at different embryological stages was inconclusive as the expression of the housekeeping gene, 18 S, also altered (increased) during development. Raw CT results are presented (Supplementary Information File 1) and, although not quantitative, the data indicate that both the 5αR genes are expressed in embryos at a detectable level by stage 3 (Gastrula).

**Figure 2:**
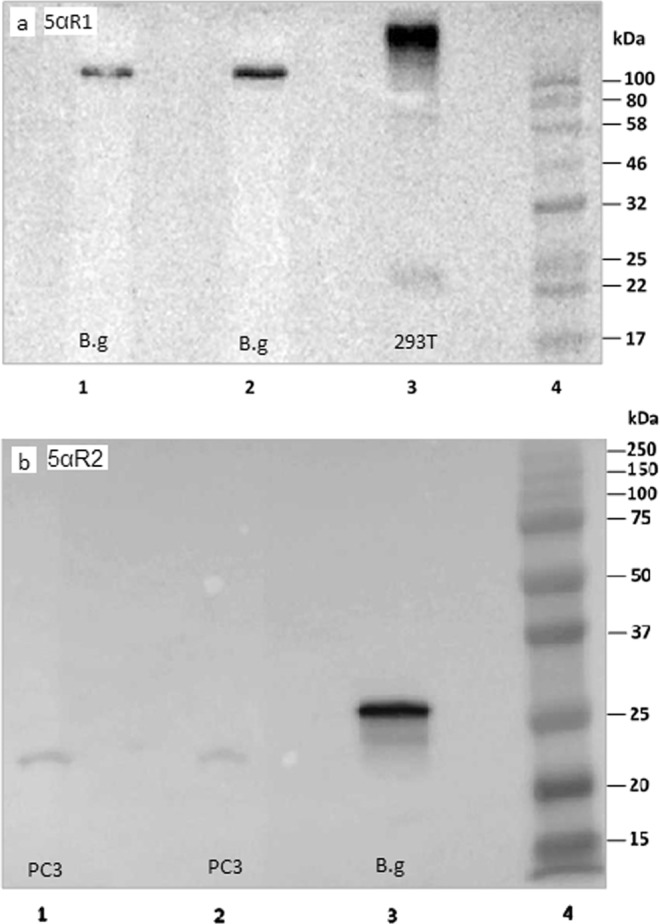
*B. glabrata* embryo lysate reacts with both 5αR1 and 5αR2 antibodies in Western blot assays, (**a**) bands can be seen for both SRD5A1 transfected 293 T cell lysate (Abnova, 10 µl) in lane 3 and *B. glabrata* embryo lysates in lanes 1 & 2 (30 µg protein per lane), (**b**) *B. glabrata* embryo lysate produces a strong reaction with human 5αR2 antibodies in lane 3 (30 µg protein per lane), human PC3 prostate cells lysate (included as a ‘positive’ response marker) is also visible but fainter in lanes 1 & 2 (170 µg protein per lane). The total protein content of both snail embryo and PC3 cell culture lysates were quantified using the Bradford assay. For both blots 10 µg of protein ladder was used per lane, (blot a, 10 µg Color Prestained Protein Standard, New England Bio-Labs, and blot b, 10 µg Precision Plus Protein™ Dual Colour Standards, Bio-rad). Images of gels have been cropped to focus on the sample bands. In each gel, only 3 samples (as described) were run. Blots for reactivity with SRD5A1 and SRD5A2 were run on separate gels and occasions. Images captured of full-length gels with additional exposures can be seen in Supporting Information Figs S1 and S2.

Western Blots were performed to determine if 5αR proteins could be detected in *B. glabrata* embryos. As both antibodies used here were raised against vertebrate (human) 5αR1 or 5αR2 (not gastropod or *B. glabrata* specific), protein lysates from a 5αR1 transfected human cell line (5αR1 293 T) and human immortalised prostate cells (PC3) were included as ‘positive’ controls for 5αR1 and 5αR2, respectively. Both 5αR antibodies (5αR1 and 5αR2) were reactive to the *B. glabrata* embryo samples (Fig. 2), suggesting both 5αR1 and 5αR2 are present during *B. glabrata* development, however, the strongest band was seen against antibody raised against 5αR2 (Fig. 2). Indeed, the total protein concentration (calculated in the Bradford assay) needed to elicit a reaction was much lower in *B. glabrata* embryo extract (30 µg total protein) than from human PC3 prostate cell line (170 µg total protein), suggesting 5αR2-like proteins are highly expressed in *B. glabrata* embryos (Fig. 2b, lane 3).

The predicted molecular weight of the human 5αR2 is 28.4 kilodaltons (kda), whereas the *B. glabrata* 5αR2 is slightly larger at 32.8 kda. These predicted molecular weights are generally in accordance with the bands seen in Fig. 2b produced with antibody raised against 5αR2. However, in Fig. 2a the bands of both species are much larger than those predicted for 5αR1 (human 5αR1 29.4 kda and *B. glabrata* 5αR1 30.9 kda). Larger size bands can occur if proteins are glycosylated or bear other post-translational modifications. Potential glycosylation sites were found in the sequences, and may reflect why the protein sizes are different to those expected in Fig. 2a.

### Induction of banana-shaped shell phenotype by pharmaceutical 5αR inhibitors

**Figure 3:**
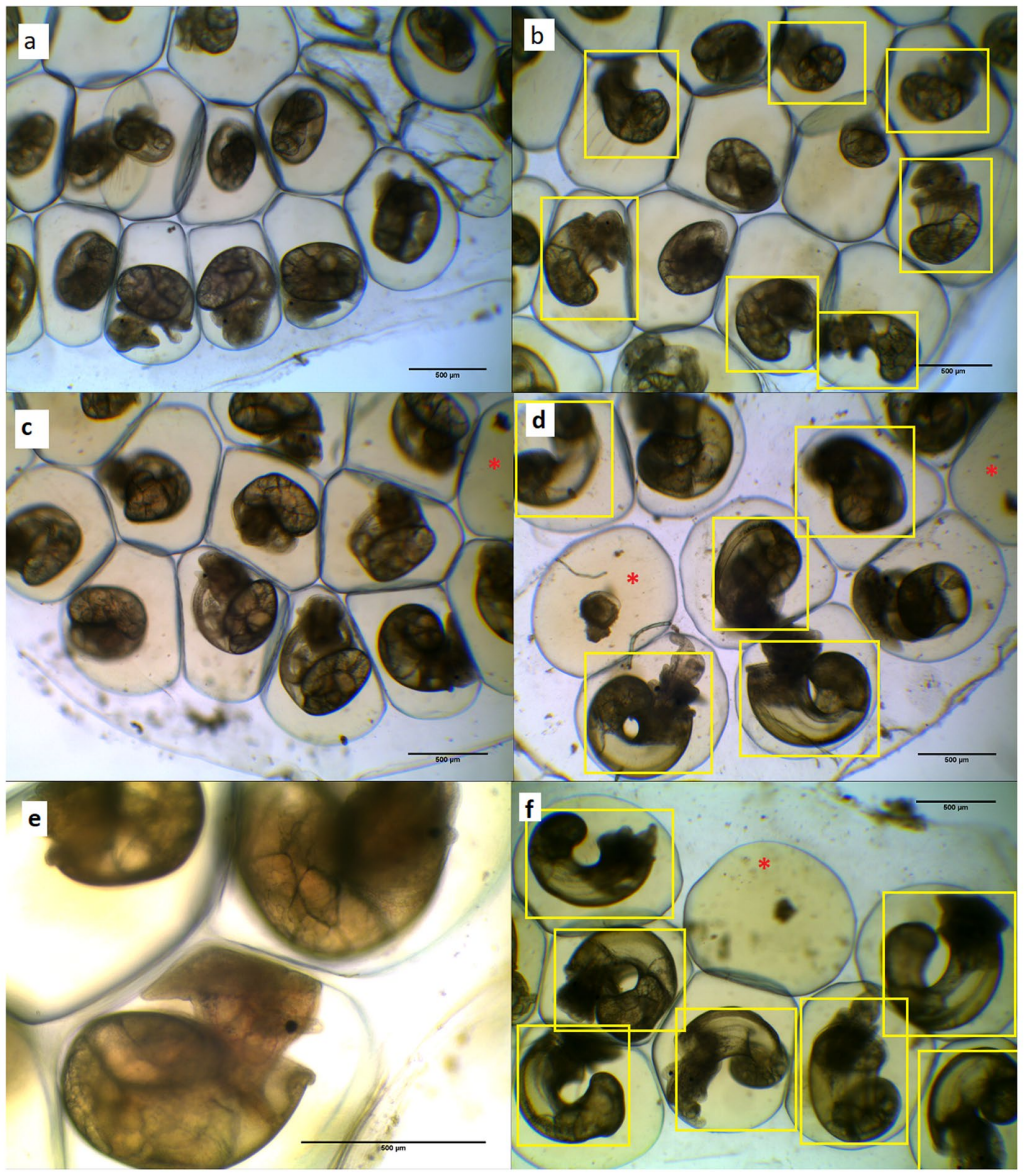
Developmental exposure to 5αR inhibitor dutasteride (DUT) elicits an elongated shell phenotype first observable under a light microscope at the ‘hippo’ stage, 4 days post fertilisation (dpf), compared to control or solvent control embryos. Photomicrographs (**a**,**c**,**e**) are solvent control *B. glabrata*, with normal compact curled shells (**a**) at 4 dpf, (**c**) at 5 dpf and (**e**) at 6 dpf. Photomicrographs (**b**,**d**,**f**) are *B. glabrata* exposed to DUT displaying an elongated banana-shaped phenotype (**b**) at 4 dpf at 100 µg/L DUT, (**d**) at 5 dpf at 100 µg/L DUT and (**f**) at 6 dpf at 160 µg/L DUT. Yellow boxes highlight individuals with elongated shells, which become more apparent as the snails continue to grow. The red asterisk indicates dead/halted development embryos.

**Figure 4:**
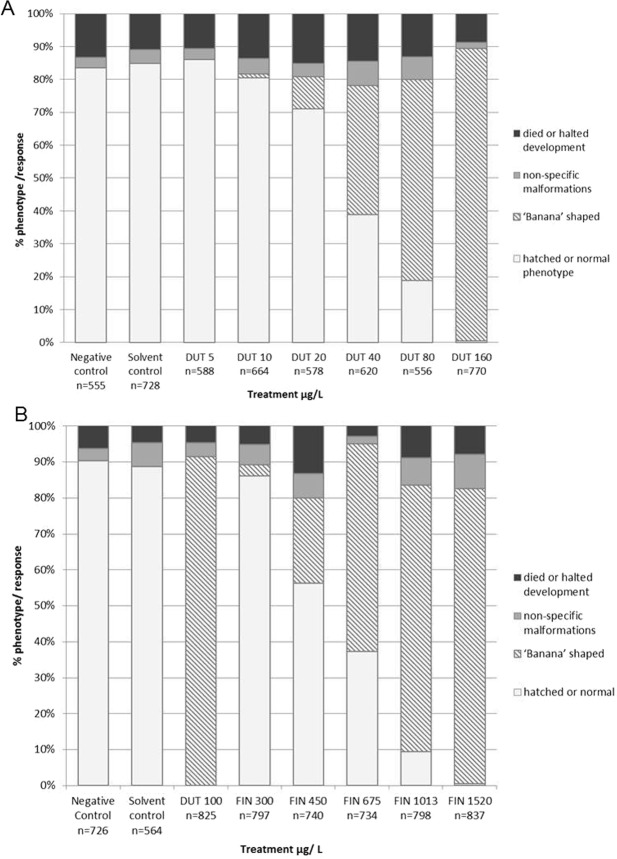
Both pharmaceutical 5α-reductase inhibitors induced a dose-dependent increase in the banana-shaped phenotype (hatched bars) when exposed during embryo development. (**A**) dutasteride (DUT) was more potent than (**B**) finasteride (FIN). Each compound’s dosing series comprised of four independent experiments; each with six ‘replicate’ egg masses per treatment. Dutasteride and finasteride dosing concentrations are in µg/L. Negative control (dilution water only) and solvent control (DMF 0.01% v/v) were included in each independent experiment to verify conditions were adequate for normal development and that the addition of carrier solvent did not negatively impact development. For the finasteride experiments, a positive control of dutasteride (100 µg/L) was also included to verify the banana-shaped phenotype could be elicited. The total number of embryos exposed to each treatment is given as n = x underneath the corresponding bar.

Both pharmaceutical 5αR inhibitors (dutasteride; DUT and finasteride; FIN) induced a strong and highly reproducible phenotypic response in the developing *B. glabrata* embryos (Figs 3, 4 and Supporting Information Table S3).

In four independent experiments, DUT induced an elongated banana-shaped shell phenotype in a dose-dependent manner. The average minimum banana-shaped phenotype response (1% of embryos had banana-shaped shell phenotype) was observed at a dose of 10 µg/L, and the average maximum response (89% of embryos had banana-shaped shell phenotype) at the highest tested dose of 160 µg/L DUT. The banana-shaped shell phenotype became significantly elevated compared to the solvent control at 40 µg/L DUT (P < 0.0001) and higher concentrations (Fig. 4). Finasteride (FIN) also induced the banana-shaped phenotype and its effects were also dose-dependent, although it was less potent than DUT, with the average (based on four independent experiments) minimum response (3% banana-shaped) at 300 µg/L and the maximum (82% banana-shaped) at 1520 µg/L (Fig. 4). FIN significantly induced the banana-shaped phenotype at 450 µg/L FIN (P < 0.0001) and above. In both the DUT and FIN experiments, the number of embryos classed as ‘normal’ was inverse to the banana-shaped phenotype and was significantly reduced in a dose-dependent manner. The percentage of other malformations or embryos that died/did not develop remained stable across the experiments and there was no significant effect (P > 0.05) of DUT or FIN concentration for these endpoints.

At 100 µg/L DUT, ≥80% of the embryos exhibited the banana-shaped phenotype. This DUT concentration was included in all other experiments testing different compounds, as a positive control and elicited significant (P < 0.0001) induction of banana-shaped phenotypes in each instance.

### Comparison of the sensitivity of various species to pharmaceutical 5αR inhibitors

Compared to other organisms, the response of *B. glabrata* to DUT (statistically significant ≥40 µg/L) or FIN (statistically significant ≥450 µg/L) seemed to be as sensitive, or perhaps more sensitive, although endpoints may differ. In the one example of pharmaceutical inhibition of plant 5αR/DET2, Cotton (*Gossypium hirsutum* L.) fibre cell growth was inhibited by FIN at 32 µM to 159 µM (32 µM = 11.9 mg)^42^; more than 20 times higher concentrations than required for *B. glabrata* disruption.

In aquatic vertebrates, Margiotta-Casaluci *et al*.^29^ found impacts on reproduction and gonad histopathology in adult fathead minnow exposed to DUT at 32 and 100 µg/L, and no significant effects at 10 µg/L. Lee *et al*.^44^ also conducted adult exposures to FIN in another fish species, the Japanese medaka. At the medium and high doses tested (500, 5000 µg/L) male fish showed gonad maldevelopment, degeneration, and Leydig cell hyperplasia^44^. The lower dose of 50 µg/L FIN did not induce significant effects^44^. In humans, DUT is known to disrupt the action of both 5αR forms (5αR1 and 5αR2) more effectively than FIN. Therefore, it seems as with humans and fish, *B. glabrata* phenotypic disruption was more sensitive to DUT than FIN.

Comparisons of developmental effects in other animal species are less clear. Developmental endpoints have not been the specific focus of any other 5αR-inhibitor study. In the medaka study by Lee *et al*.^44^, the F1 offspring from the 5000 μg/L FIN exposure showed increased time to hatch, decreased hatching rate, increased deformities in hatched larvae (pericardial oedema), and increased mortality after hatch^44^. These increased mortalities and deformities continued until two weeks after hatching, after which those that survived ‘appeared normal in behaviour and appearance’ although data were not shown^44^. Margiotta-Casaluci *et al*.^29^ also observed reduced fathead minnow embryo hatchability in 100 µg/L DUT exposed F1 embryos if the eggs were also exposed to 100 µg/L DUT during development/hatching period, but not if eggs were kept in control water after being laid by DUT exposed parents. These developmental effects were not specifically attributed to 5αR disruption, although the highly lipophilic nature of the drug was considered as a possible reason for this toxicity^29^. In comparison, developmental exposure in the frog (*Xenopus tropicalis*) to FIN 25 µM (~9.3 mg/L) induced shifts in sex ratio and intersex gonads in the resulting adults^45^ and exposure to 100 µM FIN reduced 5αR gene expression (whole-mount *in situ* hybridization) in the tadpoles^46^; however no overt toxicity or developmental malformations were reported in either of these studies.

### The specificity of the 5αR inhibition banana-shaped phenotype in *B. glabrata*

Eight other compounds, including three other pharmaceutical steroidogenic enzyme inhibitors (Galeterone (GAL); CYP17A1 inhibitor, Trilostane (TRI); 3β-HSD inhibitor, Atorvastatin (ATO); HMG-CoA reductase inhibitor), one 5β-reductase inhibitor (Chenodeoxycholic acid (ChenA)), a mutagen (Benzoquinoline (BENZO)), and an anti-inflammatory chemical (γ-linolenic acid (y-LIN) were tested in the embryonic *B. glabrata* assay to assess whether the banana-shaped phenotype was specific to 5αR inhibitors, or if it was a general enzyme-inhibition or toxicity effect. None of the other compounds tested elicited the banana-shaped phenotype (Supporting Information Table S4).

No significant phenotypic or mortality effects were seen with TRI, ATO, y-LIN, ChenA or GAL at concentrations up to 3000 µg/L and 1500 µg/L (GAL was insoluble at 3000 µg/L) (Supporting Information Table S3). BENZO was toxic at 3000 µg/L and induced elevated embryo malformations (21%) and significant mortalities (79%, P < 0.0001). At 1500 µg/L BENZO mortalities levels were comparable to controls (8%), however, general malformations were still elevated (19%) resulting in significantly less normal hatchlings (P < 0.0001, Supporting Information Table S4). None of the malformations induced in the BENZO treatment resembled the banana-shaped phenotype.

Dorsomorphin (DORS) was included as it had previously been reported to induce ‘immature shell-shape’ or cone-shaped (non-coiling) phenotypes in the gastropod *Lymnaea stagnalis* (at 0.5 and 1 µM, depending on exposure window)^47^. However, no banana-shaped phenotype or non-coiling embryos were observed in any of the DORSO concentrations we tested (Supporting information Table S3). In our test system, DORSO was extremely toxic to *B. glabrata* with 94% and 91% mortality at 400 (1 µM) and 300 µg/L respectively. Mortality was reduced to 37% at 200 µg/L (0.5 µM). Our embryo mortality rates are comparable to those reported by Shimizu *et al*.^47^ in *L. stagnalis* exposed to DORS at blastula (98.7% at 1 µM and 25.8% at 0.5 µM). Indeed, Shimizu *et al*.^47^ did not find specific shell malformation frequently, with a maximum of 10 embryos out of 265 (3.8%) and 6 out of 360 (1.7%) exhibiting shell malformations when exposed from 2-cell stage or blastula to 0.5 µM DORS, higher doses mainly caused mortality. In the later stage exposures percentage induction rates were even lower (<1% trochophore and veliger, 0% gastrula) and the shell phenotype was not dose-dependently induced^47^.

Similarly, SB431542 was included as a test compound due to its reported effect on shell chirality in gastropods^48^. SB431542 has very low solubility in water. Attempts to use the same carrier solvent concentration as for the other test compounds (i.e. 0.01% v/v) resulted in SB431542 precipitating out of solution. Therefore a higher solvent concentration (1% v/v DMF) was tested for this compound (same % of solvent (DMSO) as used by Grande & Patel^48^). However, this higher percentage of the solvent caused 100% mortality in the solvent control, 5 µM (1.9219 mg/l) and 10 µM (3.8439 mg/l) SB431542, and in the 100 µg/L DUT. Control snails, i.e. those without solvent, in the same experiment developed normally. The experiment showed obvious solvent-induced mortality, it was repeated once to confirm the outcome and then not assessed again. In terms of percentage altered shell phenotype with SB431542, Grande & Patel^48^ also had low frequencies, with a total of 4 out of 138 (2.9%) embryos having modified shells at 5 µM SB431542 and 3 out of 86 (3.5%) embryos at 10 µM SB431542. In these SB431542 exposures mortality was high; 63% at 5 µM and 91.9% at 10 µM^48^.

When comparing the banana-shaped response seen in our work to the ‘immature shell-shape’, cone-shape, or loss of shell chirality response seen by Shimizu *et al*.^47^ and Grande & Patel^48^, the major difference observed relates to toxicity. The main impact of both Dorsomorphin and SB431542 on gastropod embryogenesis was lethality, not phenotypic changes to shell development. Whereas, in the experiments reported here, mortality in the 5αR inhibitor exposures (DUT and FIN) was comparable to controls. This suggests that the impact of 5αR disruption in the snail is targeted to a specific developmental role, rather than global cell or tissue function. This is consistent with the generally non-lethal effects of FIN and DUT in people, rodents, fish, frogs and plants^28,29,42,44,45,49^ and 5αR2/DET2 mutations in people and plants^22,39^, whereas, bone morphogenetic protein (BMP) and Transforming Growth Factor-β (TGF-β) (disrupted by DORS and SB431542) are known to be crucial in embryogenesis as well as development and maintenance of all organ systems.

In summary, none of the other compounds tested (pharmaceutical enzyme inhibitors or experimental chemicals) induced the banana-shaped phenotype in developing *B. glabrata* embryos (Supporting information Tables 3). Only the potent pharmaceutical 5αR inhibitors elicited the developmental effect of shell malformation. Which suggests that the 5αR inhibitors were specifically impacting a particular enzyme vital to *B. glabrata* morphological development.

### Induction of banana-shaped phenotype in a second freshwater gastropod species

**Figure 5:**
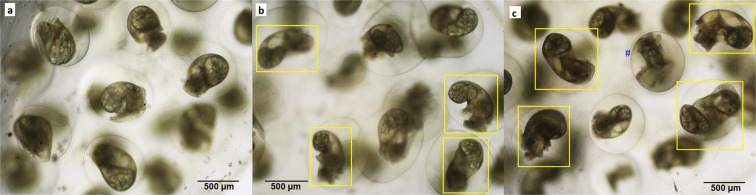
Developmental exposure to dutasteride induces the banana-shaped phenotype in a second freshwater gastropod species, *Physella acuta*, at a comparable frequency to *B. glabrata*. All images are taken at 5 dpf. Photomicrograph (**a**) solvent control *P. acuta*, with normal compact shells, (**b**) 100 µg/L DUT exposed *P. acuta* displaying elongated banana-shaped shells, (**c**) 200 µg/L DUT exposed *P. acuta* displaying a more acute phenotype (longer shell elongation). A non-specific malformation is indicated by blue #. Yellow boxes highlight individuals with elongated shells.

To test whether the pharmaceutical 5αR inhibited phenotype was species-specific, a small experiment was conducted with a second freshwater gastropod species, *Physella acuta*. *P. acuta* embryos were developmentally exposed to solvent control, 100 and 200 µg/L DUT. This experiment induced strikingly similar results to *B. glabrata*, with 71.2 and 82.6% of the embryos developing the elongated banana-shaped phenotype in the 100 and 200 µg/L DUT respectively (Fig. 5, Supporting information Table S5). Both DUT doses tested produced significant induction (P < 0.0001) of banana-shaped snails compared to the solvent control, with the reciprocal reduction in normal shaped embryos. As seen with *B. glabrata* no significant effects of DUT dose were found for the number of non-specific malformations (P > 0.9999, P = 0.8770, respectively) or deaths (P = 0.9282, P > 0.9999, respectively).

### Implications for naturally occurring non-coiled gastropod shells

A number of gastropods, such as those within the Vermetidae^50^ and the Caecidae families^51^ produce shells that are elongated in shape compared to typical coiled gastropod shells. Variation in coiling can also be seen within freshwater gastropods, and there is some debate about whether these curious findings are distinct species or if the unusual shell growth is a sporadic aberrant phenotype^52^. Recent investigations into a corkscrew-like shell of the typically planispiral-shaped *Gyraulus* sp., discovered in a remote Tibetan lake, suggest these phenotypes are the result of as-yet-unknown ecological stressor rather than speciation^52^. We cannot say whether the phenotypes observed with 5αR pharmaceutical inhibition in our laboratory are related to the unusually-shaped *Gyraulus* shells, or whether the elongated shell shapes in vermetids and caecids are due to variations in the endocrine pathway of these molluscs. However, future research on molluscan endocrinology and shell development may shed light on these remarkable specimens.

## Conclusions

Here we demonstrate that pharmaceutical 5αR inhibitors induced a strong and highly reproducible phenotypic response in developing *B. glabrata* and *P. acuta* embryos that is not associated with toxicity. We have also confirmed that both 5αR homologues identified in *B. glabrata* are expressed during embryonic development and that antibodies raised against human 5αRs proteins are reactive in *B. glabrata* embryo extracts; this strongly suggests that pharmaceutical 5αR inhibitors could be acting on *B. glabrata* 5αR enzymes to cause this phenotypic disruption. In adult *B. glabrata*, 5αRs transcripts have been detected in mantle tissue^32^. This is of note as molluscan shells are formed from secretory cells in the mantle^21^, providing a possible link between 5αR and shell formation.

Molluscs, like other invertebrates, do not possess the cholesterol side-cleavage enzymes involved in the early stages of vertebrate sex hormone steroidogenesis^38^. This lack of *de novo* synthesis of androgens strongly implies novel substrates for 5αR exist in gastropods. Therefore, further work is required to investigate the potential role of 5αR’s in molluscan biology, including what the possible substrates and products of 5αR enzymes could be.

Primarily, this research highlights that assumptions on gene function based on knowledge gained from vertebrates may not hold for other phyla, and that there are still many fundamental knowledge gaps to be filled for molluscs including steroidogenesis and associated endocrine processes. Now we have more powerful molecular tools to start these investigations, a new era of research focusing on this large, diverse and ecologically important group of animals has the potential to shed light on the evolution of endocrine system, support new developments in aquaculture and agriculture, as well as to inform chemical testing regulation to protect mollusc populations in the wild.

## Methods

### Ethics statement

Ethical approval from the Brunel University London Animal Welfare Ethics Review Board (AWERB) and the UK Home Office was not required for this study as gastropod molluscs, such as *B. glabrata*, are not protected by the UK Animal [Scientific Procedures] Act 1986. Likewise, ethics approval was not required for research on *P. acuta* within Australia. Nevertheless, as the work was *in vivo*, this manuscript was prepared with ‘Animal Research: Reporting *In Vivo* Experiments’ (ARRIVE) guidelines^53^ in mind.

### Test organism

The tropical freshwater pulmonate snail *B. glabrata* was selected as a test species due to its relatively well-documented physiology and recent genome sequencing^32^. In comparison to other species with published genomes such as the Pacific oyster (*Crassostrea gigas*), Owl limpet (*Lottia gigantea*) and California two-spot octopus (*Octopus bimaculoides*), *B. glabrata* is easily housed, has a short generation time, reproduces regularly, and can easily complete its lifecycle in the laboratory as it lacks a planktonic life-stage. It also lays large numbers of eggs within flat translucent egg masses which are easily visualised under the microscope for embryo observation.

*B. glabrata* (BB02 strain; obtained originally from The Natural History Museum, London), were maintained in glass aquaria supplied with de-chlorinated tap water. Adult snails, for egg production, were maintained in de-chlorinated tap water in flow-through aquaria at 27 °C and fed *ad libitum* three times a week with Tetramin fish flakes.

Adult *P. acuta* were collected from a suburban garden pond in Brisbane, Australia, and housed in 500 mL glass beakers filled with pond water at 27 °C. Water was exchanged weekly and adults were fed with boiled spinach and lettuce twice per week. Egg-laying behaviour was initiated after several days, and egg masses were removed from the sides of beakers using a plastic scraper.

### Identification of 5α-reductase transcripts in *B. glabrata* embryos

Total RNA was extracted from staged embryo samples (4 hr, 4–24 hr, gastrula, trochophore, veliger and hippo, n = 3 egg masses at each stage) using the RNeasy Fibrous Tissue Mini Kit (Qiagen) according to the manufacturer’s protocol. This kit includes DNAse treatment to eliminate genomic DNA contamination. cDNA synthesis was carried out in 20 µl reactions, using 40 ng total RNA from individual egg mass, using the Superscript III cDNA synthesis kit (Invitrogen, Life Technologies, Carlsbad. USA) with 5 μM of a custom oligo (dTAP) primer (TGACTCGAGTCGACATCGAT^54^) following the manufacturer’s instructions. Residual RNA was removed by adding 1 μl of RNase H (2 U/μl) to the reaction and incubating it at 37 °C for 20 min. Specific primers were designed from the sequenced fragments of 5aR1 and 5aR2 (Supporting Information Table S1) using Primer3. 18 S primers (18S-F: CGCCCGTCGCTACTATCG and 18S-R: ACGCCAGACCGAGACCAA^54^). PCRs of 20 µl contained Power SYBR® Green Master Mix according to the manufacturers protocol (Applied Biosystems), 10 pmol of each primer and 1 µL of 1/5 dilution of cDNA synthesized from individual egg masses. PCR cycling conditions were: 50 °C for 2 min, 95 °C for 10 min, then 40 cycles of 95 °C for 30 sec, X °C for 1 min, using the CFX96 Real-time PCR Detection system (Biorad), where X is the optimized annealing temperature for each primer pair (18 S- 55 °C, 5aR1- 59 °C and 5aR2- 57 °C). A dissociation curve was generated in each case to check that only a single band was amplified. qPCRs were performed in triplicate for each egg mass.

### 5α-reductase proteins in *B. glabrata* embryos

To detect 5αR1 and 5αR2 protein expression in *B. glabrata* embryos, replicates of 100 hippo stage embryos (4-days post-fertilisation) were dissected from their egg masses and homogenised in 100 µL of cold RIPA buffer (Sigma Aldrich, UK) with a cocktail of protease inhibitor (Roche). The embryo lysates were then centrifuged and the pellet discarded. The protein concentrations of the resulting lysates were determined by Bradford assay^55^. Samples were diluted with Laemmli buffer and heated at 95 °C for 5 minutes. For each embryo lysate, 10 µl of the sample containing 30 µg of total protein was loaded into the polyacrylamide gel (12% Mini-PROTEAN® TGX™ Precast Protein Gels, Bio-Rad). A protein molecular weight marker (10 µl, Precision Plus Protein™ Dual Colour Standards, Bio-Rad or Color Prestained Protein Standard, New England Bio-Labs) and positive control samples (detailed below) were also loaded on to the gel. The proteins in these samples were separated by gel electrophoresis (running buffer Tris/Glycine/SDS) based on molecular weight (SDS-PAGE). Immediately after electrophoresis, gels were blotted onto Immun-Blot PVDF membranes (Bio-Rad, UK) at 4 °C for 1 hour at 100 V in a blotting chamber filled (Bio-Rad) with transfer buffer (25 mM Tris, 192 mM glycine, 20% (v/v) methanol). To reduce non‐specific binding, membranes were blocked with 5% fat-free milk powder in 0.2% TBS-tween for 1 hour with rocking at room temperature. The membranes were then incubated with the primary antibody (either: anti SRD5A2, anti-SRD5A1, Abcam, UK) at 1:1000 dilution in 0.2% TBS-Tween with rocking overnight at 4 °C. Membranes were then washed in 0.2% TBS-tween three times for ten minutes each, and incubated with secondary antibody (anti-rabbit IgG HRP-linked antibody, Cell Signalling Technology) at 1:5000 dilution in 0.2% TBS-tween containing 5% fat-free milk at room temperature with rocking for 1 hour. After the secondary antibody incubation, membranes were washed again three times for ten minutes each in 0.2% TBS-tween. The blots were incubated with luminol-based Western blotting substrates (Amersham ECL Prime Western Blotting Detection Reagent, GE Healthcare). The blots were then imaged using a Molecular Imager (VersaDoc 4000MP, Bio-Rad).

For anti-SRD5A1 blot, an SRD5A1 transfected 293 T cell lysate (Abnova) was used as a positive comparison sample. For anti-SRD5A2 blot, human immortalised prostate cells PC3 (lysed in 100 µl of RIPA buffer/cocktail, centrifuged and treated as above) was included as a positive control.

### Test compounds

All compounds were purchased in pure form (>90%) from Sigma Aldrich UK. Concentrated stock solutions were prepared in HPLC grade N, N-dimethylformamide (DMF) 99% stored at 4 °C; see Supporting Information Table S2 for details of selected compounds.

Chemical exposures in water were prepared by spiking 100 mL of de-chlorinated aquarium water with a given concentrated stock solution or DMF. Unless otherwise stated chemical treatments and solvent control contained DMF at 0.01% v/v (at the suggested maximum solvent concentration of 0.01% v/v OECD limit for aquatic vertebrates OECD 2000 and gastropods OECD 2010). The control treatment contained de-chlorinated aquarium water only. All chemical concentrations are nominal as we did not measure the chemicals in the water.

### Embryo exposures

For each experiment *B. glabrata* egg masses were collected from parental breeding tanks on the day they were laid, all embryos were at blastula stage^56^ when dosing commenced.

Individual experiments included both control and solvent controls (six replicates of each) to monitor the development and viability of the eggs. Each treatment, within an experiment, consisted of six independent ‘replicate’ egg masses (~20–60 eggs/mass); one per well of a six-well plate (Nunc). All control, solvent control and compound exposed eggs were kept in 5 mL of dosing media in the dark and incubated at 27 °C for 7 days. At 27 °C *B. glabrata* normally hatch between 5–10 days post oviposition under our laboratory conditions. On the first day of the exposure each egg mass was photographed under x2 and x4 magnification and imageJ 1.5 (‘multi-point’ tool) was used to count the number of embryos per mass. Embryos were monitored for development and mortality throughout the experiment.

Preliminary experiments with the potent 5αR inhibitor, DUT (100 µg/L), elicited a strong phenotypic response in the developing *B. glabrata*. At hippo stage^56^, embryos had unusual body-plan most obvious in their elongated shell development (Fig. 3). Shells did not curl in the normal way i.e. tightly in the rams-horn shape of the species, and were described as banana-shaped (Fig. 3).

On day six of the experiment each embryo/larva was classed as either: ‘dead’ (not developed or halted development), banana-shaped phenotype, ‘non-specific malformation’ or ‘normal’ (e.g. hatched and/or developed with usual shell phenotype). For graphical representation responses were then expressed as a percentage of the total number of embryos in the mass (at the start of the exposure).

Four other vertebrate steroidogenic enzyme inhibitors (Finasteride (FIN); 5αR inhibitor, Galeterone (GAL); CYP17A1 inhibitor, Trilostane (TRI); 3β-HSD inhibitor, Atorvastatin (ATO); HMG-CoA reductase inhibitor), one 5β-reductase inhibitor (Chenodeoxycholic acid (ChenA)), an oxidoreductive enzyme inhibitor (Azelaic acid (AzA)), a mutagen (Benzoquinoline (BENZO)), and an anti-inflammatory chemical (γ-linolenic acid (y-LIN), were also tested to assess the specificity of the phenotype. Experiments for banana-shaped phenotypic responses were conducted at doses considered to be biologically active in other test systems (*in vitro* and/or *in vivo*, Supporting Information Table S2). Dorsomorphin (bone morphogen protein inhibitor) and SB431542 (inhibitor of transforming growth factor-β type I activin receptor-like kinase) were also investigated as these compounds have previously been reported to induce non-coiling shell phenotypes in gastropods (Grande & Patel^48^, Shimizu *et al*.^47^).

For each compound, at least two independent experiments were conducted to investigate the reproducibility of the response. Each experiment included a positive (DUT 100 µg/L) and negative (solvents and water only) controls.

The dose-response experiments were also conducted for compounds that induced the banana-shaped phenotype. These were conducted to investigate potency and to identify if the compound produced ‘classic’ dose-dependent activity i.e. highest dose elicits the highest activity. For the dosing series 5, 10, 20, 40, 80 and 160 µg/L DUT and 300, 450, 675, 1013 and 1520 µg/L FIN were tested.

As the purpose of the experiments was to assess phenotypic endpoints, rather than overt toxicity, egg masses that contained more than 50% of dead or non-developing embryos after 24hrs were considered as being of poor quality and removed from the analysis.

Due to SB431542 having very low solubility in water, this compound was eventually tested using a higher concentration of solvent, 1% v/v DMF (as earlier experiments resulted in compound dropping out of solution). An equivalent solvent concentration across all treatment groups (including the solvent control and the positive control (DUT)) was adjusted to 1% DMF for comparison.

To test whether the pharmaceutical 5αR inhibited phenotype was species-specific or not, a smaller experiment with DUT was also conducted with another freshwater gastropod species, *Physella acuta*, in a different laboratory. The treatments were carried out as per the *B. glabrata* exposures outlined above, except that three replicate *P. acuta* egg masses per treatment were exposed to either solvent control (DMF at 0.01% v/v), 100 µg/L DUT, or 200 µg/L DUT, and incubated at 27 °C for five days. Embryos were monitored for development and mortality throughout the experiment. At 5 dpf the embryos were observed and the number of normal shaped, banana-shaped, non-specific malformed and dead embryos were scored and representative photos captured. The DUT and dosing stocks for this experiment were purchased and prepared independently from those used in the previous experiments with *B. glabrata*.

### Analysis and statistics

The multiple comparisons tests, two-way ANOVA followed by Dunnett’s, were used to analyse the different phenotypes (normal and banana-shaped) and developmental outcomes (non-specific malformation, died) in response to the different chemical treatments. The solvent control group was used as the factor to compare all other treatments in each experiment. For all tests α was set at 0.05, above which the null hypothesis, that there has been no effect of treatment, was accepted. Analyses were conducted in GraphPad Prims 7.

## Supplementary information

Supplementary information

## Data Availability

All data generated or analysed during this study are included in this published article (and its Supplementary Information Files). Data produced during pilot studies linked to this work are available from the corresponding author on reasonable request.
