# Supplementary material for: Early embryonic exposure of freshwater gastropods to pharmaceutical 5-alpha-reductase inhibitors results in a surprising open-coiled “banana-shaped” shell
Source: Sci Rep. 2019 Nov 11;9:16439. doi: 10.1038/s41598-019-52850-x (PMC6848481; doi:10.1038/s41598-019-52850-x)

Supporting Information

Early embryonic exposure of freshwater gastropods to pharmaceutical 5α-reductase inhibitors results in a surprising open-coiled "banana-shaped" shell

Alice Baynes[1]*, Gemma Montagut Pino[1,2], Giang Huong Duong[1], Anne E Lockyer[1], Carmel McDougall[3], Susan Jobling[1], Edwin J Routledge[1]

[1]Institute of Environment, Health and Societies, Brunel University London, Uxbridge, UB8 3PH, United Kingdom

[2]Centre for Obesity Research, Division of Medicine, University College London (UCL), 5 University Street, London, WC1E 6JF, United Kingdom

[3]Australian Rivers Institute, Griffith University, 170 Kessels Road, Nathan QLD 4111, Australia

* Corresponding author

E-mail: Alice.Baynes@Brunel.ac.uk

Table S1. QPCR primers used for 5αR1 and 5αR1 expression profiling in *Biomphalaria glabrata* embryos

gene	Primer
BG18SQSP1	CGCCCGTCGCTACTATCG
BG18SQASP1	ACGCCAGACCGAGACCAA
qPCR5aR1Fb	GGCCTGAGTGTATGCGTTC
qPCR5aR1Rb	CAACACAGCAGGGTAGTTCTTG
5aR2F	CATCATCAACAGATGGGCAGA
5aR2R	CACAAACTCAAACAAGCCTCC

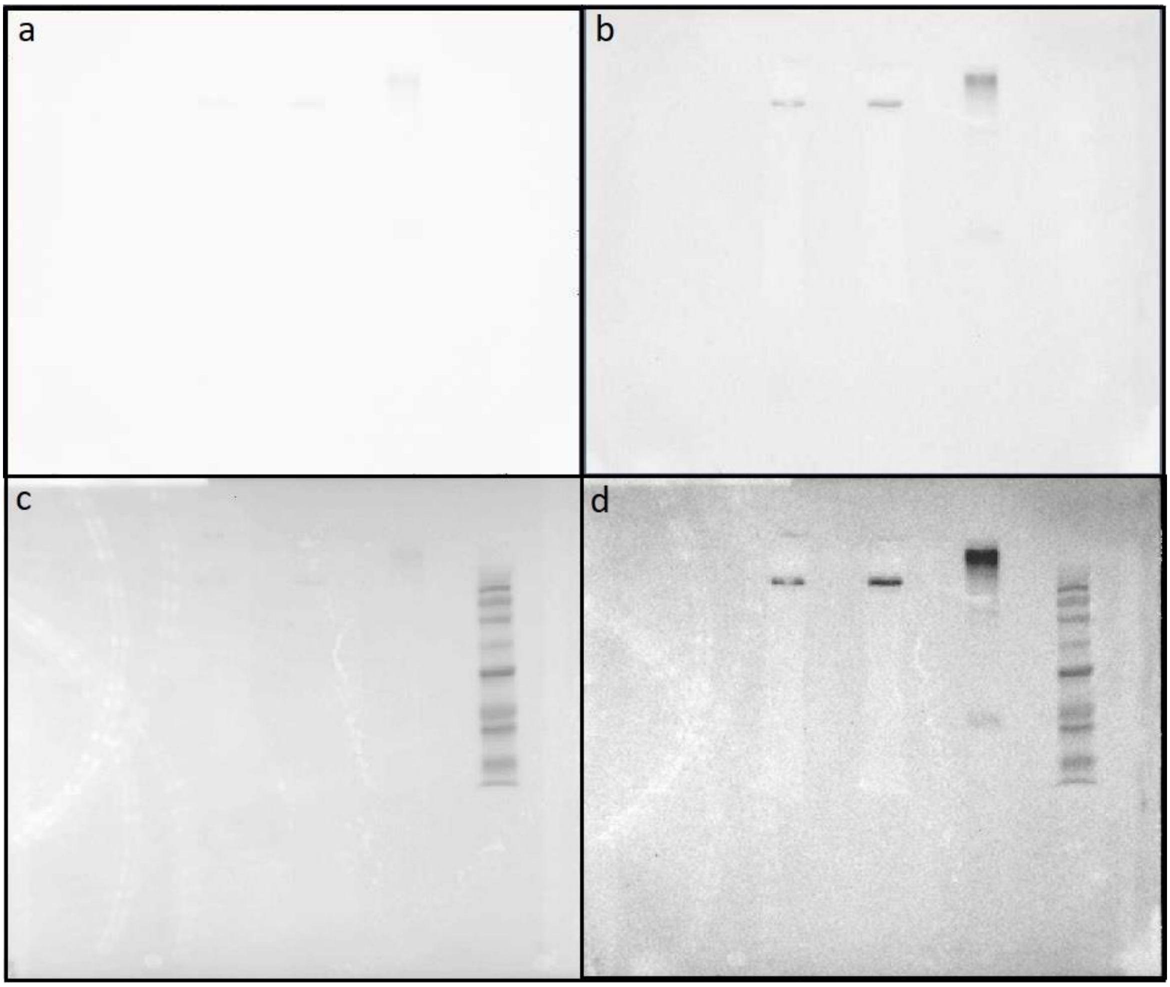

Figure S1. *B. glabrata* embryo lysate and SRD5A1 transfected 293T cell lysate (Abnova, 10 µl) reactions with 5αR1 antibodies in Western blot assay. Here we show multiple images, full-length gels and multiple exposures, of the same western blot gel in Figure 2a main text. Image S1a and S1b are prior to ladder overlay, S1c and S1d are images after ladder overlay.

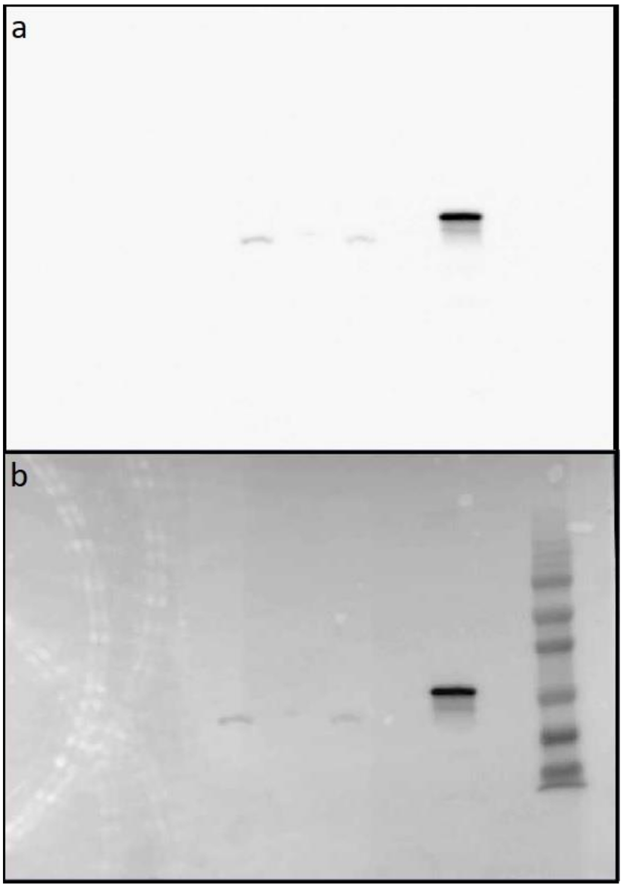

Figure S2. *B. glabrata* embryo lysate and human PC3 prostate cells lysate reactions with 5αR2 antibodies in Western blot assays. Here we show multiple images, the full-length gel and additional exposure, of the same western blot gel in Figure 2b main text. Image S2a is prior to ladder overlay, S2b is after ladder overlay.

Table S2. Compounds tested on *B. glabrata* embryos to investigate impact of 5αR-inhibition during development and specificity of the 'banana-phenotype'.

**Compound name**	**CAS**	**Molecular mass**	**Primary mode of action**	**Compound type**	**Concentrations tested**	**Previous test species, biological endpoint and concentration ranges**
Dutasteride (DUT)	164656-23-9	528.53	Potent duel 5αR1 and 5αR2 inhibitor	Pharmaceutical	5-150 µg/L	*Pimephales promelas* 10-100 µg/L reproduction/gametogenesis[1]
Finasteride (FIN)	98319-26-7	372.55	5αR2 inhibition	Pharmaceutical	300-1520 µg/L	*Oryzias latipes* 50-5000 µg/L reproduction/gametogenesis [2] *Xenopus tropicalis* 25 µM (9.3 mg/L) gametogenesis [3] and 100 µM (37.26 mg/L) inhibition of 5αR gene expression [4] *Gossypium hirsutum* 32-159 µM fiber cell growth [5]
γ-linolenic acid (γ-LIN)	506-26-3	278.43	Possible anti-inflammatory, weak inhibitor of androgen steroidogenesis (used to treat skin disorders/eczema)	Polyunsaturated fatty acid	3000 µg/L	*In vitro* LNCaP prostate cells, 5-50µM conversion of T to DHT [6]
Chenodeoxycholic acid (ChenA)	474-25-9	392.57	5β-reductase inhibitor	Bile acid	3000 µg/L	*Xenopus tropicalis* 100 µM (37.26 mg/L) inhibition of 5αR gene expression [4]
Benzo[h]quinoline (BENZO)	85-02-9	179.22	mutagen	Experimental compound	1500, 3000 µg/L	*Salmonella typhimurium* (TA 100 of histidine-dependent strain) 0-200 µg/plate [8]
Galeterone (GAL)	851983-85-2	388.25	inhibitor of CYP17A1 (CYP450c17) (*steroidogenesis inhibitor*)	Pharmaceutical	1500 µg/L	Human *in vitro* IC50 300 nM CYP17 inhibition [9]
Trilostane (TRI)	13647-35-3	329.43	inhibitor of 3 β-hydroxysteroid dehydrogenase (3-β-HSD) (*steroidogenesis inhibitor*)	Pharmaceutical	3000 µg/L	*Pimephales promelas* 60-1500 µg/L reproduction/gametogenesis [10]
Atorvastatin (ATO)	134523-03-8	558.64	inhibitor of HMG-CoA reductase (*steroidogenesis inhibitor*)	Pharmaceutical	3000 µg/L	*Danio rerio* ~0.53 µg/g fish cholesterol, cortisol, testosterone and estradiol levels [11]
Dorsomorphin (DORS)	866405-64-3	399.49	inhibitor of Bone morphogenetic protein (BMP) signaling	Experimental compound	200-400 µg/L (0.5µM-1µM)	*Lymnaea stagnalis* 0.5 µM – 10 µM non-mineralised or 'cone-like' non-coiling shell [12]
SB431542	301836-41-9	384.39	inhibits transforming growth factor beta (TGF-β)	Experimental compound	1.9, 3.8 mg/L (5µM, 10µM)	*Biomphalaria glabrata* 5 µM, 10µM 'long' non-coiling shell/ loss of shell chirality [13]

Table S3. Both pharmaceutical-5αR-inhibitors elicited the banana-shaped phenotype in *Biomphalaria glabrata* embryos in a dose-dependent manner. Summary of development and hatching data for dutasteride and finasteride dosing series, each compound's dosing series comprised of four independent experiments; each with six replicate egg masses per treatment. Negative control (dilution water only) and solvent control (DMF 0.01% v/v) were included in each independent experiment to verify conditions were adequate for normal development and that the addition of carrier solvent did not negatively impact development. For the finasteride experiments a positive control of dutasteride (100 μg/L) was also included to verify the 'banana' phenotype could be elicited in each experiment.

**Experiment: Dutasteride dosing series**					
**Chemical treatment and concentration**	**Total number of embryos exposed**	**Percentage hatched or normal**	**Percentage 'Banana' shaped**	**Percentage non-specific malformations**	**Percentage died or halted development**
**Negative control**	555	84	0	3	13
**Solvent control**	728	85	0	4	11
**DUT 5 μg/L**	588	86	0	3	11
**DUT 10 μg/L**	664	80	1	5	14
**DUT 20 μg/L**	578	71	10	4	15
**DUT 40 μg/L**	620	39	39	7	15
**DUT 80 μg/L**	556	19	61	7	13
**DUT 160 μg/L**	770	0.5	89.6	1.7	8.2
**Experiment: Finasteride dosing series**					
**Chemical treatment and concentration**	**Total number of embryos exposed**	**Percentage hatched or normal**	**Percentage 'Banana' shaped**	**Percentage non-specific malformations**	**Percentage died or halted development**
**Negative control**	726	90	0	4	6
**Solvent control**	564	89	0	7	5
**Positive control (DUT 100 μg/L)**	825	0	91	4	5
**FIN 300 μg/L**	797	86	3	6	5
**FIN 450 μg/L**	740	56	24	7	13
**FIN 675 μg/L**	734	37	58	2	3
**FIN 1013 μg/L**	798	9	74	8	9
**FIN 1520 μg/L**	837	0.5	82	9.6	7.9

Table S4. None of the other pharmaceutical steroidogenic enzyme inhibitors (non 5αR) elicited the banana-shaped phenotype in *Biomphalaria glabrata* embryos. Summary development and hatching date for Galeterone; GAL (inhibitor of CYP17A1), Chenodeoxycholic acid; CHEN (5β-reductase inhibitor), Trilostane; TRIL (inhibitor of 3 β-hydroxysteroid dehydrogenase), Atorvastatin; ATRO (inhibitor of HMG-CoA reductase), Benzo[f]quinolone; BENZO (Mutagen; Experimental compound - not pharmaceutical), γ-linolenic acid; LINO (anti-inflammatory Polyunsaturated fatty acid), Dorsomorphin; DORSO (bone morphogen protein inhibitor), and SB431542 (inhibitor of transforming growth factor-β type I activin receptor-like kinase) embryo exposures. Each compound was tested in at least two independent experiments, with six replicate egg masses per treatment. Negative control (dilution water only), solvent control (DMF 0.01% v/v) and a positive control of dutasteride (100 µg/L) were included in each independent experiment to verify conditions were adequate for normal development, that the addition of carrier solvent did not negatively impact development and to verify the 'banana' phenotype could be elicited, respectively. *Solvent concentrations in the SB431542 experiments were 1% v/v DMF due to the insolubility of SB431542, 100% mortality were associated with this level of DMF.

**Experiment: Galeterone and Chenodeoxycholic acid**					
**Chemical treatment and concentration**	**Total number of embryos exposed**	**Percentage hatched or normal**	**Percentage 'Banana' shaped**	**Percentage non-specific malformations**	**Percentage died or halted development**
**Negative control**	728	90	0	6	4
**Solvent control**	804	86	0	10	4
**Positive control (DUT 100 µg/L)**	767	0	82	8	10
**GAL 1500 µg/L**	561	87	0	6	7
**CHEN 3000 µg/L**	627	89	0	5	6
**Experiment: Trilostane and Atorvastatin**					
**Chemical treatment and concentration**	**Total number of embryos exposed**	**Percentage hatched or normal**	**Percentage 'Banana' shaped**	**Percentage non-specific malformations**	**Percentage died or halted development**
**Negative control**	682	90	0	4	6
**Solvent control**	487	94	0	4	2
**Positive control (DUT 100 µg/L)**	517	0	91	3	7
**ATOR 3000 µg/L**	581	88	0	9	3
**TRIL 3000 µg/L**	542	94	0	2	4
**Experiment: Benzo[f]quinolone and γ-linolenic acid**					
**Chemical treatment and concentration**	**Total number of embryos exposed**	**Percentage hatched or normal**	**Percentage 'Banana' shaped**	**Percentage non-specific malformations**	**Percentage died or halted development**
**Negative control**	418	86	0	4	10
**Solvent control**	457	92	0	3	5
**Positive control (DUT 100 µg/L)**	398	4	78	4	14
**BENZO 1500 µg/L**	204	72	0	12	16
**BENZO 3000 µg/L**	531	0	0	24	76
**LINO 3000 µg/L**	579	89	0	7	4
**Experiment: Dorsomorphin**					
**Chemical treatment and concentration**	**Total number of embryos exposed**	**Percentage hatched or normal**	**Percentage 'Banana' shaped**	**Percentage non-specific malformations**	**Percentage died or halted development**
**Negative control**	405	92	0	4	4

**Solvent control**	412	91	0	3	6
**Positive control (DUT 100 µg/L)**	394	0	85	6	9
**DORSO 200 µg/L**	443	55	0	8	37
**DORSO 300 µg/L**	430	8	0	1	91
**DORSO 400 µg/L**	429	5	0	2	94
**Experiment: SB431542**					
**Chemical treatment and concentration**	**Total number of embryos exposed**	**Percentage hatched or normal**	**Percentage 'Banana' shaped**	**Percentage non-specific malformations**	**Percentage died or halted development**
**Negative control**	365	84	0	9	7
**Solvent control***	395	0	0	0	100
**Positive control* (DUT 100 µg/L)**	243	0	0	0	100
**SB431542 1900 µg/L***	353	0	0	0	100
**SB431542 3800 µg/L***	359	0	0	0	100

Table S5. The banana-shaped phenotype is elicited in second freshwater gastropod species *Physella acuta* when embryos are exposed during development to 5αR inhibitor dutasteride. Experiment consisted of three replicate *P. acuta* egg masses per treatment: solvent control (DMF 0.01% v/v), 100 µg/L and 200 µg/L DUT. Embryos were at blastula stage at the start of dosing, phenotypic/developmental endpoints were recorded on 5 dpf.

**Chemical treatment and concentration**	**Total number of embryos exposed**	**Percentage normal**	**Percentage 'Banana' shaped**	**Percentage non-specific malformations**	**Percentage died or halted development**
**Solvent control**	103	95.6	0.0	1.5	3.0
**DUT 100 µg/L**	105	19.8	71.2	1.8	7.2
**DUT 200 µg/L**	112	8.3	82.6	5.6	3.5

References

1. Margiotta-Casaluci, L., Hannah, R. E. & Sumpter, J. P. Mode of action of human pharmaceuticals in fish: The effects of the 5-alpha-reductase inhibitor, dutasteride, on reproduction as a case study. *Aquat. Toxicol.* **128,** 113–123 (2013).

2. Lee, M. R., Loux-Turner, J. R. & Oliveira, K. Evaluation of the 5 alpha-reductase inhibitor finasteride on reproduction and gonadal development in medaka, Oryzias latipes. *Gen. Comp. Endocrinol.* **216,** 64–76 (2015).

3. Duarte-Guterman, P. *et al.* The Aromatase Inhibitor Fadrozole and the 5-Reductase Inhibitor Finasteride Affect Gonadal Differentiation and Gene Expression in the Frog Silurana tropicalis. *Sex. Dev.* **3,** 333–341 (2010).

4. Bissegger, S. & Langlois, V. S. Steroid 5-reductases are functional during early frog development and are regulated via DNA methylation. *Mech. Dev.* **141,** 14–24 (2016).

5. Luo, M. *et al.* GhDET2, a steroid 5 alpha-reductase, plays an important role in cotton fiber cell initiation and elongation. *PLANT J.* **51,** 419–430 (2007).

6. LIANG, T. M. & LIAO, S. S. INHIBITION OF STEROID 5-ALPHA-REDUCTASE BY SPECIFIC ALIPHATIC UNSATURATED FATTY-ACIDS. *Biochem. J.* **285,** 557–562 (1992).

7. STAMATIADIS, D., BULTEAUPORTOIS, M. C. & MOWSZOWICZ, I. INHIBITION OF 5-ALPHA-REDUCTASE ACTIVITY IN HUMAN-SKIN BY ZINC AND AZELAIC ACID. *Br. J. Dermatol.* **119,** 627–632 (1988).

8. KUMAR, S. *et al.* MUTAGENICITY AND TUMORIGENICITY OF DIHYDRODIOLS, DIOL EXPOSIDES, AND OTHER DERIVATIVES OF BENZO(F)QUINOLINE AND BENZO(H)QUINOLINE. *CANCER Res.* **49,** 20–24 (1989).

9. Handratta, V. D. *et al.* Novel C-17-heteroaryl steroidal CYP17 inhibitors/antiandrogens: Synthesis, in vitro biological activity, pharmacokinetics, and antitumor activity in the LAPC4 human prostate cancer xenograft model. *J. Med. Chem.* **48,** 2972–2984 (2005).

10. Villeneuve, D. L. *et al.* Effects of a 3 beta-hydroxysteroid dehydrogenase inhibitor, trilostane, on the fathead minnow reproductive axis. *Toxicol. Sci.* **104,** 113–123 (2008).

11. Al-Habsi, A. A., Massarsky, A. & Moon, T. W. Exposure to gemfibrozil and atorvastatin affects cholesterol metabolism and steroid production in zebrafish (Danio rerio). *Comp. Biochem. Physiol. B-BIOCHEMISTRY Mol. Biol.* **199,** 87–96 (2016).

12. Shimizu, K., Sarashina, I., Kagi, H. & Endo, K. Possible functions of Dpp in gastropod shell formation and shell coiling. *Dev. Genes Evol.* **221,** 59–68 (2011).

13. Grande, C. & Patel, N. H. Nodal signalling is involved in left-right asymmetry in snails. *Nature* **457,** 1007–1011 (2009).

Supplementary File 1.

Early embryonic exposure of freshwater gastropods to pharmaceutical 5α-reductase inhibitors results in a surprising open-coiled “banana-shaped” shell.

Alice Baynes[1], Gemma Montagut Pino[1,2], Giang Huong Duong[1], Anne E Lockyer[1], Carmel McDougall[3], Susan Jobling[1], Edwin J Routledge[1]

[1]Institute of Environment, Health and Societies, Brunel University London, Uxbridge, UB8 3PH, United Kingdom

[2]Centre for Obesity Research, Division of Medicine, University College London (UCL), 5 University Street, London, WC1E 6JF, United Kingdom

[3]Australian Rivers Institute, Griffith University, 170 Kessels Road, Nathan QLD 4111, Australia

Sample	18S	5aR1b	5aR2
NTC	37.23		38.33
Bg1a	30.44		
Bg1a	30.53	38.68	
Bg1a	30.38		39.24
Bg1b	30.41	38.71	38.72
Bg1b	30.49		39.33
Bg1b	30.30		38.18
Bg1c	31.01	36.00	
Bg1c	31.13		
Bg1c	30.58		
Bg2a	29.17	36.71	
Bg2a	29.07	36.30	
Bg2a	28.97	35.78	39.91
Bg2b	24.44	38.01	39.42
Bg2b	24.65		38.41
Bg2b	24.24	38.63	38.99
Bg2c	28.03	39.87	
Bg2c	27.84		38.97
Bg2c	27.44		39.03
Bg3a	21.56	36.07	37.21
Bg3a	21.29	34.80	38.73
Bg3a	21.28	35.88	36.39
Bg3b	22.34		37.22
Bg3b	22.32		36.91
Bg3b	21.87		38.37

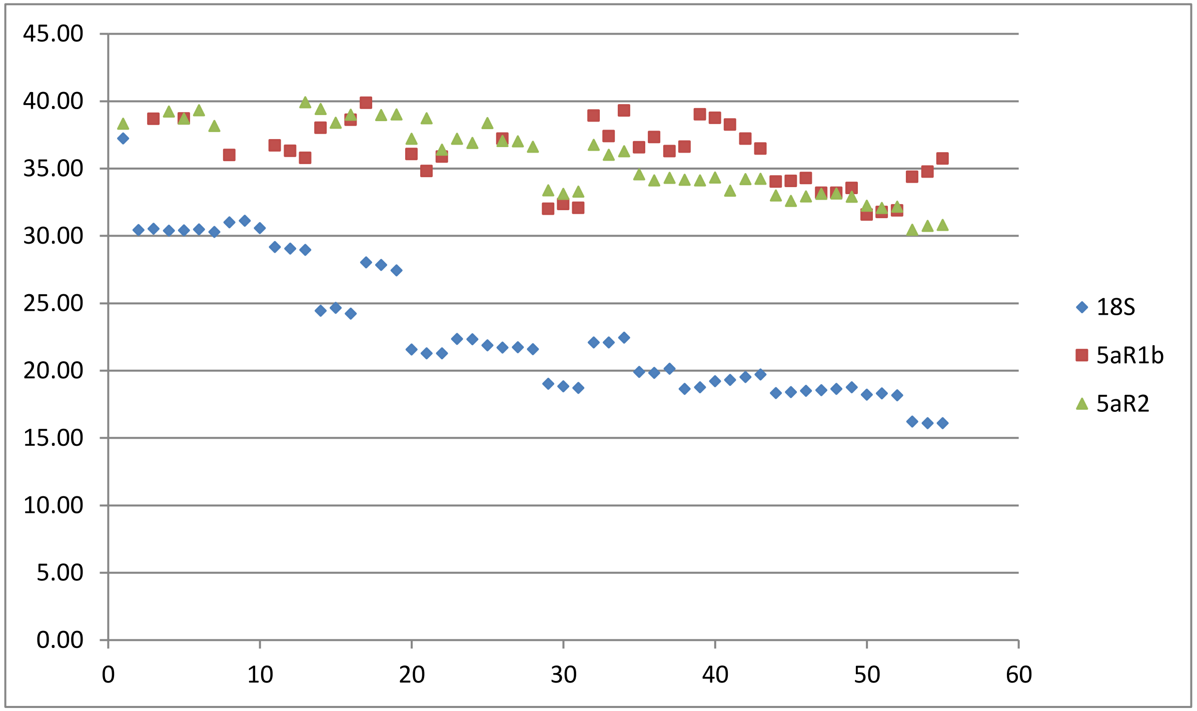

Bg3c	21.71	37.21	37.04
Bg3c	21.73		37.01
Bg3c	21.60		36.62
Bg4a	19.04	32.01	33.39
Bg4a	18.83	32.36	33.13
Bg4a	18.71	32.07	33.28
Bg4b	22.08	38.93	36.76
Bg4b	22.08	37.41	36.02
Bg4b	22.44	39.30	36.28
Bg4c	19.89	36.56	34.56
Bg4c	19.84	37.33	34.13
Bg4c	20.14	36.28	34.31
Bg5a	18.64	36.62	34.17
Bg5a	18.77	39.03	34.11
Bg5a	19.21	38.76	34.34
Bg5b	19.31	38.25	33.36
Bg5b	19.53	37.22	34.22
Bg5b	19.71	36.48	34.25
Bg5c	18.33	34.02	33.01
Bg5c	18.40	34.07	32.61
Bg5c	18.51	34.28	32.94
Bg6a	18.56	33.19	33.12
Bg6a	18.65	33.19	33.14
Bg6a	18.77	33.54	32.91
Bg6b	18.21	31.59	32.25
Bg6b	18.31	31.76	32.08
Bg6b	18.17	31.88	32.18
Bg6c	16.22	34.39	30.46
Bg6c	16.11	34.77	30.75
Bg6c	16.11	35.75	30.81

Sample	Mean 18S	5aR1b	5aR2	sd 18S	5aR1b	5aR2
Bg1a	30.45	38.68	39.24	0.074		
Bg1b	30.40	38.71	38.74	0.100		0.580
Bg1c	30.91	36.00		0.291		
Bg2a	29.07	36.27	39.91	0.100	0.468	
Bg2b	24.44	38.32	38.94	0.209	0.433	0.509
Bg2c	27.77	39.87	39.00	0.304		0.043
Bg3a	21.37	35.58	37.45	0.162	0.683	1.187
Bg3b	22.18		37.50	0.269		0.769
Bg3c	21.68	37.21	36.89	0.845	0.290	
Bg4a	18.86	32.15	33.27	0.163	0.185	0.133
Bg4b	22.20	38.55	36.35	0.207	1.003	0.375
Bg4c	19.96	36.72	34.34	0.159	0.547	0.218
Bg5a	18.87	38.14	34.21	0.300	1.319	0.120
Bg5b	19.52	37.32	33.94	0.202	0.888	0.503
Bg5c	18.41	34.13	32.85	0.093	0.140	0.216
Bg6a	18.66	33.31	33.06	0.106	0.201	0.130
Bg6b	18.23	31.74	32.17	0.072	0.147	0.087
Bg6c	16.15	34.97	30.67	0.062	0.700	0.185

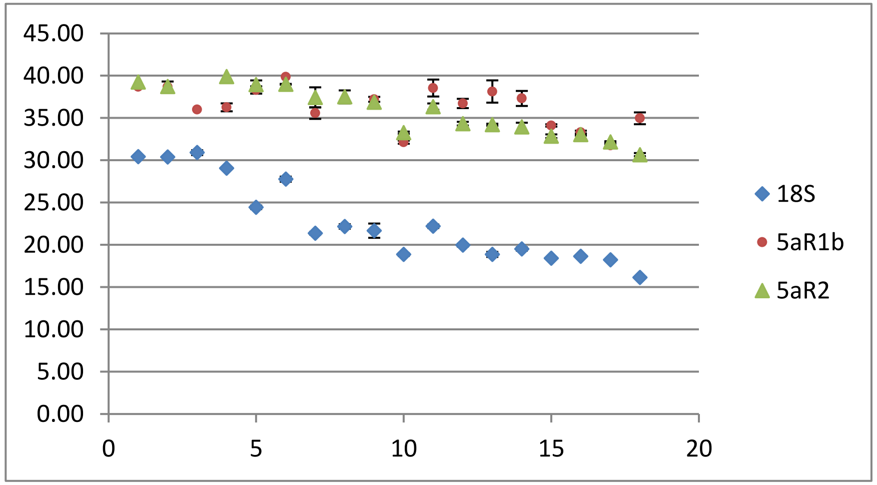

stage	18S	5aR1b	5aR2	18S	5aR1b	5aR2
1	30.59	37.80	38.87	0.288972	1.554633	0.535743
2	27.09	37.55	39.12	2.075102	1.559	0.504677
3	21.74	35.99	37.28	0.386676	0.987487	0.774141
4	20.34	35.81	34.65	1.483539	2.914662	1.374789
5	18.93	36.53	33.67	0.514903	2.001638	0.68277
6	17.68	33.34	31.97	1.165465	1.445268	1.050914

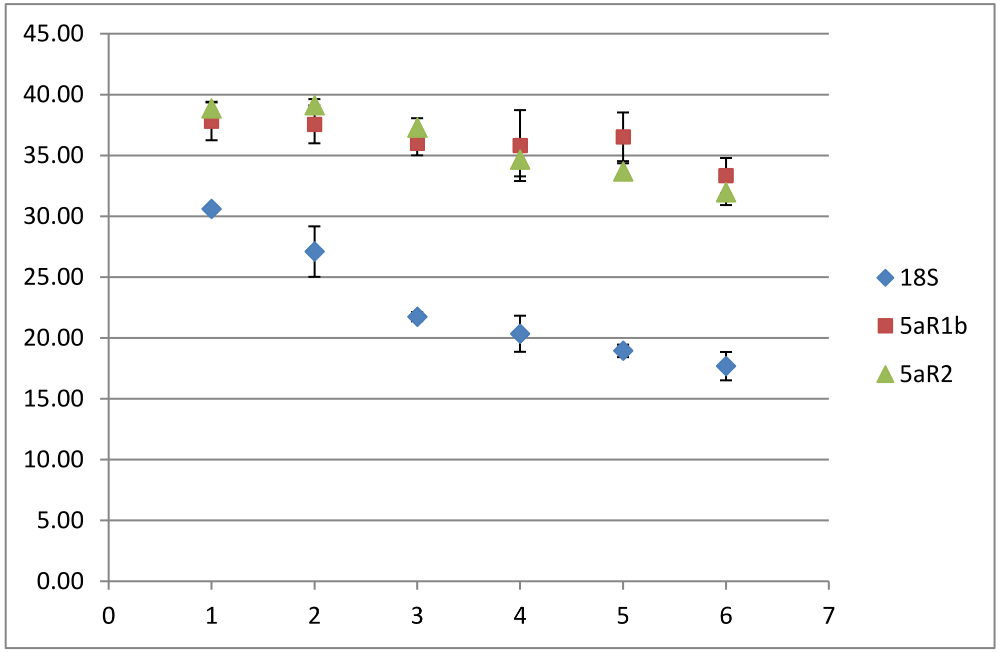

Supplement: Supplementary file 1 — Supplementary information [file 41598_2019_52850_MOESM1_ESM.pdf]
